# Annotations

**Published:** 1892-09-10

**Authors:** 


### Annotations.

Medical Sickness and Annuity.

The *Insurance Observer* takes us a little sceptically to task for a notice which appeared in the columns of The Hospital a week or two ago of the annual report of the Medical Sickness and Annuity Fund. The Fund, which has considerable resources, and which confers unusual benefits upon its sick policy holders and annuitants calls itself a Life Assurance Association as well as a Sickness and Annuity Fund. It is this claim to be a Life Office, together with the exceedingly low working expenses rate which makes the *Observer's* watch-dog bark. The facts are, however, very simple. The premium income of the Medical Sickness and Annuity Fund is considerable, whilst, thanks to the honesty of its policy holders, the claims upon its benefits are by no means numerous. One result is a rapid accumulation of resources; and another, that every medical man who is a member of the Fund experiences the feeling of security against sickness which he so properly desires to have, whilst the comparatively few who become actually disabled receive benefits of a really solid and substantial kind. If to these facts be added this, that the management is both honorary and skilful, an adequate explanation is supplied. With regard to the purely life assurance part of the business, it is probable that very little of this is done, and that what is done is only for small amounts.

The Fever Hospitals Full.

The Fever Hospitals under the control of the Metropolitan Asylums Board have at length been filled to their utmost capacity. The epidemic of scarlet and other fevers shows, however, no signs of abating. We have frequently pointed out the fact, which ought to be obvious, but apparently is not, that epidemics make no attempt to regulate their activity by the convenience of municipal or other authorities. They go on their own way, regardless of every consideration of public or private happiness, and only cease when their energy is exhausted, or efficient sanitary barriers are erected across their paths. It is certainly a pity that the ordinary resources of the Metropolitan Asylums Board have come to an end, but it does not strike us as being an occasion for scolding or the tearing of hair. Rather it is an occasion for the making of practical suggestions for dealing with the present emergency, and for the pushing forward with all haste of the additional provision of a permanent kind which has already been taken in hand at Tottenham. The *Daily Chronicle* makes a suggestion which is very much to the point. Why not rent a few large untenanted houses for a time? says the *Chronicle*, and convert them into fever hospitals. They could easily be disestablished and disinfected bye-and-by when their temporary purpose has been served. This seems to us an excellent idea. Whether it be new or not is quite unimportant. It is a better idea, indeed, than that of building huts, or other costly structures of a purely extemporaneous kind. It cannot surely be proposed to sit down with folded hands and to announce to the world *a non possumus!* That would be to unconditionally throw down our arms in the presence of an enemy which might prove almost as dangerous as cholera itself. If scarlet fever patients are to be left in the homes in which they are attacked, we deliberately invite the epidemic, which is now local and partial, to become universal. Practical science feels itself ashamed at the bare suggestion of such a confession of failure. We are as much bound to effectually isolate every scarlet fever case as we should be to defend our English coast if it were attacked by a foreign enemy. The thing has got to be done, whatever method we employ, and we feel assured that the Local Government Board will by no means be content until it has made due provision for the complete isolation of every metropolitan case of infectious fever.

Milk and Scarlet Fever.

Glasgow is now struggling with an outbreak of scarlet fever, small indeed, and localised, thanks to strict precautions, but curious in its origin. Some difficulty was at first experienced in tracing whence it arose, but investigation showed that all the families affected had been supplied with milk from one dairy. The dairy has been put in quarantine, the sanitary authorities have acted promptly, and there is practically no danger of the disease becoming epidemic; but the fever remains as an instance paralleled only by the famous Hendon case of scarlet fever arising from the consumption of infected milk. Whence the infection came has not yet been shown. Possibly some milkmaid, well to all appearance, had not wholly passed the stage of desquamation when she returned to her work, and some microscopic fragments of her skin finding their way into the milk spread the taint around. But this is guess work. As a rule, any fault in milk comes out in enteric symptoms, and while most critics are disposed to wax hyper-sarcastical and suggest the adulteration of the milk with impure water, graver inquirers ask if it is not possible that the cows that supplied the milk were fed on grass fertilised by sewage. This, of course, raises a new problem, for at present the authorities of our great cities would be only too glad to know that they could employ sewage harmlessly and profitably in agriculture, instead of casting it out to poison hapless fish in sea and stream. Some analysts, however, have surmised a difference in the decomposition of milk taken from cows fed on ordinary grass and that of animals which have grazed on ground fertilised by sewage, and that the latter contains germs dangerous to health. If this be so, it seems possible that the microbes of many diseases may thus be reintroduced into human organisms, and far-reaching mischief done. It is hardly possible, dealing with germs so infinitely small and so infinitely numerous, to say with certainty what bacteria, what shreds and scraps of infectious matter, will survive exposure to the air, burial in the ground, transit through some lower animal, and re-appear as capable of mischief as before. The comma bacillus of cholera has, we know, a persistent vitality; so have some others; and while complete certainty about all is wanting, it is safe to beg that all natural manures be disinfected before use. As for the householder, his course is clear. He cannot go to see whence all his supplies come. But he can boil his milk before using it.

